# Ninjin'yoeito Alleviates Neuropathic Pain Induced by Chronic Constriction Injury in Rats

**DOI:** 10.3389/fnut.2021.525629

**Published:** 2021-02-04

**Authors:** Risa Takemoto, Seiwa Michihara, Li-Kun Han, Nina Fujita, Ryuji Takahashi

**Affiliations:** Kampo Research Laboratories, Kracie Pharma, Ltd., Toyama, Japan

**Keywords:** Ninjin'yoeito, CCI, neuropathic pain, corticosterone, BDNF, chronic pain

## Abstract

Kampo medicines are frequently used empirically to treat pain in clinical practice. Ninjin'yoeito (NYT), which is associated with few adverse effects, is often used to treat the elderly, but has not yet been examined in detail. We herein investigated the effects of NYT, at 500 and 1,000 mg/kg p.o. (NYT500/NYT1000 group) in single and repeated administrations for 14 days, on pain in rats with peripheral neuropathy induced by loose ligation of the sciatic nerve (chronic constriction injury: CCI). Untreated CCI rats given distilled water were used as a control group. To assess induced pain, the pain threshold was measured using the von Frey test. To evaluate spontaneous pain, the ground-contact area of the paw with neuropathic pain was measured using the Dynamic Weight Bearing test. Serum samples were collected after the test to elucidate the mechanism of action of NYT, and brain-derived neurotrophic factor (BDNF) and corticosterone protein levels, which have been reported to change due to chronic pain, were analyzed. After single administration of NYT, the pain threshold rose in the NYT500 and NYT1000 groups. The pain threshold tended to rise on day 14 of repeated administration in the NYT500 group (*p* = 0.08) and it significantly rose at NYT1000 group (*p* < 0.05) compared to Control group. In addition, the foot contact area increased (*p* = 0.09). Therefore, CCI-induced pain was significantly remitted and spontaneous pain was remitted after repeated administration of NYT. Serum BDNF levels were higher in untreated CCI rats than in normal rats (*p* = 0.05), but decreased after the repeated administration of NYT (NYT1000, *p* = 0.15), while serum corticosterone levels were lower (*p* = 0.12) than those in normal rats and increased after the repeated administration of NYT (NYT1000, *p* = 0.07). The blood BDNF level has been suggested to influence pain intensity. The findings demonstrated NYT effectively treats neuropathic pain, suggesting that a NYT-induced decrease in blood BDNF contributed to the mechanism of pain relief. In addition, the variation of corticosterone was observed, suggesting that normalization of responsiveness to stress by NYT contributed to the pain relief.

## Introduction

Chronic pain has been estimated to affect ~25 million individuals in Japan (1), and has been divided into two categories: inflammatory and neuropathic pain (2). Neuropathic pain is caused by a lesion or disease of the somatosensory nervous system. It reduces the quality of life of patients and is a frequently encountered condition that limits the doses and durations of treatments. A common treatment for neuropathic pain is antidepressant drugs and Ca^2+^ channel α2δ ligands; however, these are associated with adverse effects. In particular, these drugs must be administered with caution to mental disorder patients and the elderly.

Kampo medicines are frequently used empirically to treat pain in clinical practice. NYT is composed of Poria Sclerotium, Japanese Angelica Root, Rehmannia Root, Atractylodes Rhizome, Ginseng, Cinnamon Bark, Citrus Unshiu Peel, Polygala Root, Peony Root, Astragalus Root, Schisandra Fruit, and Glycyrrhiza (Table 1). NYT, which is associated with few adverse effects, is often used to treat the elderly. Mitsuhata reported the use of NYT and Yokkansan chimpi hange in pain treatments (3). Furthermore, NYT was shown to be effective for low back pain and lower abdominal pain accompanying anemia (4). In addition, it has recently been reported in a randomized controlled trial that NYT is useful to treat pain induced by an anticancer drug, oxaliplatin (5). In an animal study, both oxaliplatin-induced cold allodynia and mechanical hyperalgesia were markedly improved by NYT extract and a crude drug comprising NYT, Ginseng, suggesting that the active crude drug of NYT for pain is Ginseng. Furthermore, NYT extract, Ginseng, and Ginsenoside Rg3 inhibited oxaliplatin-induced suppression of neurite outgrowth of the primary dorsal root ganglion in a concentration-dependent manner in an *in vitro* system, identifying that ginsenoside Rg3 is one of the active ingredients (6). A rat model of chronic constriction injury (CCI) simulating symptoms of chronic nerve compression is a neuropathic pain model close to clinical cases (7, 8). It has been reported that liquiritin, an ingredient of licorice herb comprising NYT, ferulic acid, an ingredient of Angelica Sinensis Radix, and catalpol, an ingredient of Rehmanniae Radix, relieved neuropathic pain of CCI (9–11). However, the usefulness of NYT for neuropathic pain of CCI has not been investigated. Thus, in this study, we investigated whether NYT relieves CCI-induced neuropathic pain in rats.

**Table 1:** Composition (daily dose^*^) of Kampo Formula Ninjin'yoeito (NYT).

**Ingredient**		**Content (g)**
**English name**	**Latin name**	
Poria sclerotium	*Poria*	4.0
Japanese angelica root	*Angelicae Radix*	4.0
Rehmannia root	*Rehmanniae Radix*	4.0
Atractylodes rhizome	*Atractylodis Rhizoma*	4.0
Ginseng	*Ginseng radix*	3.0
Cinnamon bark	*Cinnamomi cortex*	2.5
Citrus unshiu peel	*Aurantii Nobilis Pericarpium*	2.0
Polygala root	*Polygalae Radix*	2.0
Peony root	*Paeoniae Radix*	2.0
Astragalus root	*Astragali Radix*	1.5
Schisandra fruit	*Schisandrae Fructus*	1.0
Glycyrrhiza	*Glycyrrhizae Radix*	1.0

* *Approximately 6,700 mg of dried water extract of NYT was prepared at the GMP-standardized factory of Kracie Pharma, Ltd. (Japan) based on the above described composition*.

## Materials and Methods

### Animals

Male SD rats treated with CCI surgery in the left foot at 5 weeks of age were purchased at 6 weeks of age from Japan SLC (Shizuoka, Japan). The CCI model was prepared following the method created by Bennet et al. (12). Rats were anesthetized with isoflurane, the skin was incised along the gap of the left foot femoral muscle, and the sciatic nerve was exposed and loosely ligated with 4–0 silk at 4 sites at 1-mm intervals. Lepetan (buprenorphine) (Otsuka Pharmaceutical Co., Ltd., Japan) was subcutaneously administered twice as an analgesic after surgery and the following day. They were reared in an air-conditioned animal house facility (room temperature 23 ± 2°C; reversed 12-h light/dark cycle; relative humidity 55 ± 10%) at Kampo Research Laboratories in Kracie Pharma, Ltd. Rats were housed in a sterilized metal cage with a wire mesh floor and provided with laboratory pellet chow (CE-2; Clea Japan, Inc.) and water *ad libitum*. Before experimental procedures, they were acclimated to the room for 1 week. The experimental protocol was approved by the Experimental Animal Care Committee of Kracie Pharma, Ltd.

### Drug Treatment

The dried extract powder of NYT (Lot No. 15112017) was used in the present study, and was manufactured by the GMP Pharmaceutical Factory of Kracie Pharma, Ltd. (Qingdao, China). The von Frey test was performed before NYT administration and animals with CCI which developed pain were selected (pain threshold: 6.0 g or lower). The selected CCI rats were divided into the following 3 groups: Control group, NYT 500 mg/kg treatment group (NYT500), and NYT 1,000 mg/kg treatment group (NYT1000), so as to make no significant difference in the pain threshold among the groups. Then, NYT was administered at 10 mL/kg B.W. once a day for 14 consecutive days starting on the 14th day post-surgery as follows: In the Control group, distilled water was orally administered. In the NYT500 and NYT1000 groups, NYT was suspended with distilled water before use and the specified dose was orally administered. The drug was administered by collaborators and efforts were made to ensure the experimenters were blinded in evaluation of the subsequent von Frey test and dynamic body weight bearing.

### Von Frey Test for Mechanical Allodynia

Mechanical allodynia in rats was evaluated using the von Frey test. In the von Frey test, a series of calibrated von Frey filaments (Touch-Test Sensory Evaluator, North Coast Medical, Inc., Morgan Hill, CA) with a bending force ranging between 1 and 15 g were applied to the midplantar skin of each hind paw at a rate of once per second. Specifically, the paw was stimulated 10 times at a speed of once per second in the order from the filament with the lowest strength and when escape behavior was noted once or more, the rat was judged as positive. Then, stimuli were added using the one-step weaker filament when the animal was positive and using the one-step stronger filament when the animal was negative. Stimulation was repeated until observing positive and negative responses to stimulation with 2 continuous filament types, respectively, and the filament strength to which a positive response was observed was recorded as the pain threshold. Fourteen days after the CCI surgery, the pain threshold of the left foot was measured before administration and on day 1 (single administration; 2 h after administration) and day 14 (repeated administration; the day following 13-day continuous administration) after initiation of administration. The pain threshold of the right foot without surgery before initiation of administration was recorded as the baseline.

### Dynamic Weight Bearing for Spontaneous Pain

The dynamic weight bearing test (Bioseb, Pinellas Park, FL) was performed referring to the method reported by Quandros et al. (13). Briefly, the device was constituted with a small plexiglass chamber (22.0 × 22.0 × 30.0 cm) equipped with a floor sensor including a pressure transducer. In this system, software recording the mean weight loaded by each of the fore- and hind feet in grams without interference with an analyzer was used. To perform the test, the rat was placed in the chamber and allowed to freely move for 6 min. The device was used for acclimation for 1 min followed by recording for 5 min. To support data analysis, a camera was turned toward the side of the enclosure. All movements were photographed and investigated following the position of the rat on the device by the experimenters and the foot corresponding to the pixel set recognized by the sensor was identified as the right or left foot. DWB software provides data concerning the area (in mm^2^) of the foot contacting the floor. The testis and tail were excluded from analysis. The results were presented as the area of the left foot. Fourteen days after the CCI surgery, measurement was performed before administration and on day 14 after initiation of administration (repeated administration) in each rat. The value measured in the right foot without surgery before initiation of administration was recorded as the baseline.

### Serum Analyses

Blood samples were collected immediately following the sacrifice of each rat and clotted for 30 min at room temperature before centrifuging for 20 min at 2,000 rpm. The supernatant was then collected for an enzyme-linked immunosorbent assay (ELISA). Serum was stored at −40°C until assayed for brain-derived neurotrophic factor (BDNF) and corticosterone. Serum BDNF (BDNF/proBDNF Rapid ELISA Kit; BIOSENSYS) and serum corticosterone [corticosterone (Human, Rat, Mouse) ELISA (RE52211); IBL] were measured by ELISA following the manufacturer's instructions. Normal rats given distilled water were used as a Normal group.

### Statistical Analysis

Data were expressed as the mean ± standard error of means. Significant differences were assessed by a one-way analysis of variance followed by Dunnett's test, or *post hoc* test followed by Steel test for multiple comparisons. Between normal group and control group were compared by Student's *t*-test. *P* < 0.05 were considered to be significant.

## Results

### Effects of Single Administration of NYT on CCI-Induced Mechanical Allodynia

**Figure 1:**
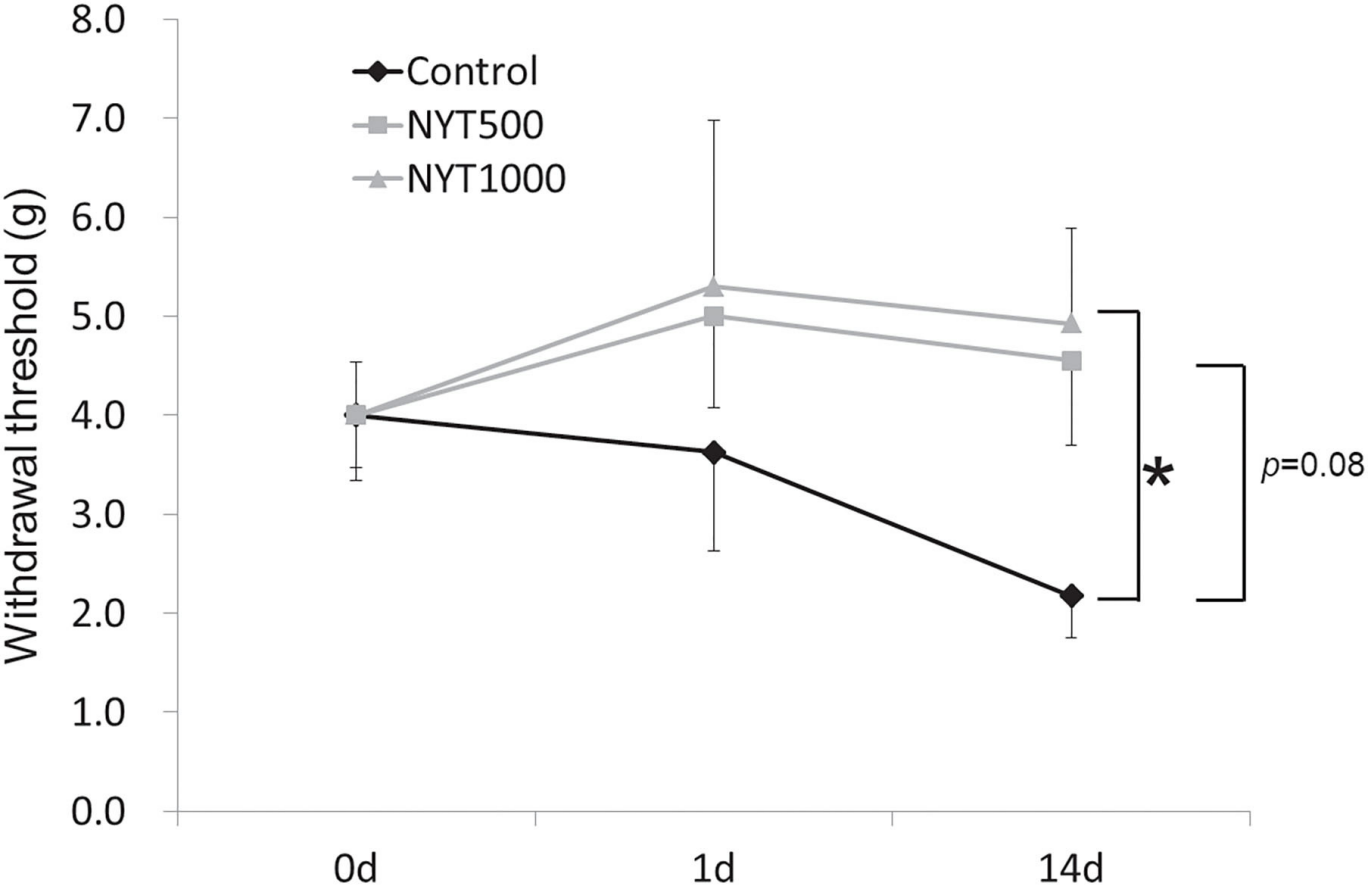
Effects of NYT on CCI-induced mechanical allodynia. Effects of NYT on CCI-induced mechanical allodynia in rats. NYT at 500 and 1,000 mg/kg p.o. in single and repeated administrations for 14 days to rats. The black object group was administered water (Control); the gray object group was administered NYT (NYT500/NYT1000). Each column shows the mean ± S.E. of 8 mice.*p < 0.05 vs. the control group, evaluated using Dunnett's test.

After single administration of NYT, the pain threshold rose in the NYT500 and NYT1000 groups (Figure 1) (Baseline: 9.1 g). Although the elevation was not significant, it was suggested that NYT exhibits an effect on pain, so administration was continued and the effect of NYT on pain by repeated administration was investigated.

### Effects of Repeated Administration of NYT on CCI-Induced Mechanical Allodynia and Spontaneous Pain

**Figure 2:**
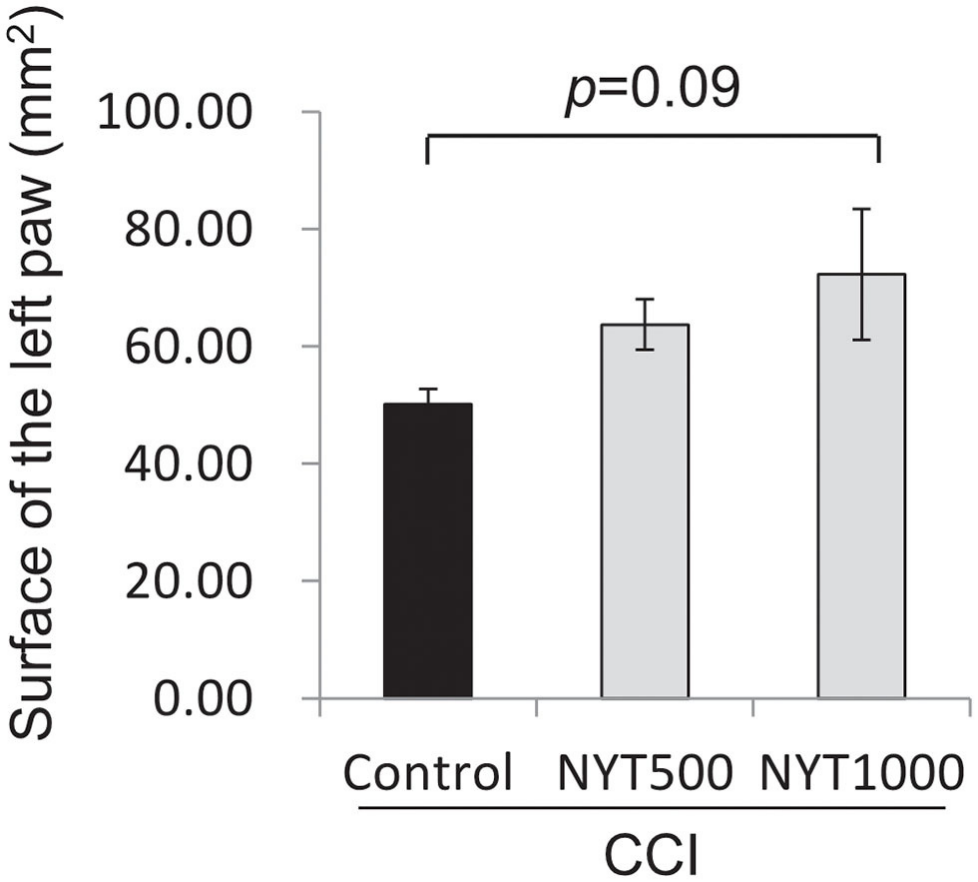
Effects of NYT on CCI-induced spontaneous pain. Effects of NYT on CCI-induced spontaneous pain in rats. NYT at 500 and 1,000 mg/kg p.o. in repeated administrations for 14 days to rats. The black column group was administered water (Control); the gray column group was administered NYT (NYT500/NYT1000). Each column shows the mean ± S.E. of 4 mice. *p*-values evaluated using Dunnett's test.

The pain threshold tended to rise on day 14 of repeated administration in the NYT500 group (*p* = 0.08, by dunnet) and it significantly rose at NYT1000 (*p* < 0.05, Dunnett's test; Figure 1). In addition, the foot contact area increased (*p* = 0.09, Dunnett's test; Figure 2) (baseline: 78.52 mm2). Therefore, CCI-induced pain was significantly remitted and spontaneous pain was remitted after repeated administration of NYT.

### Effects of NYT on BDNF Protein Expression in Serum

**Figure 3:**
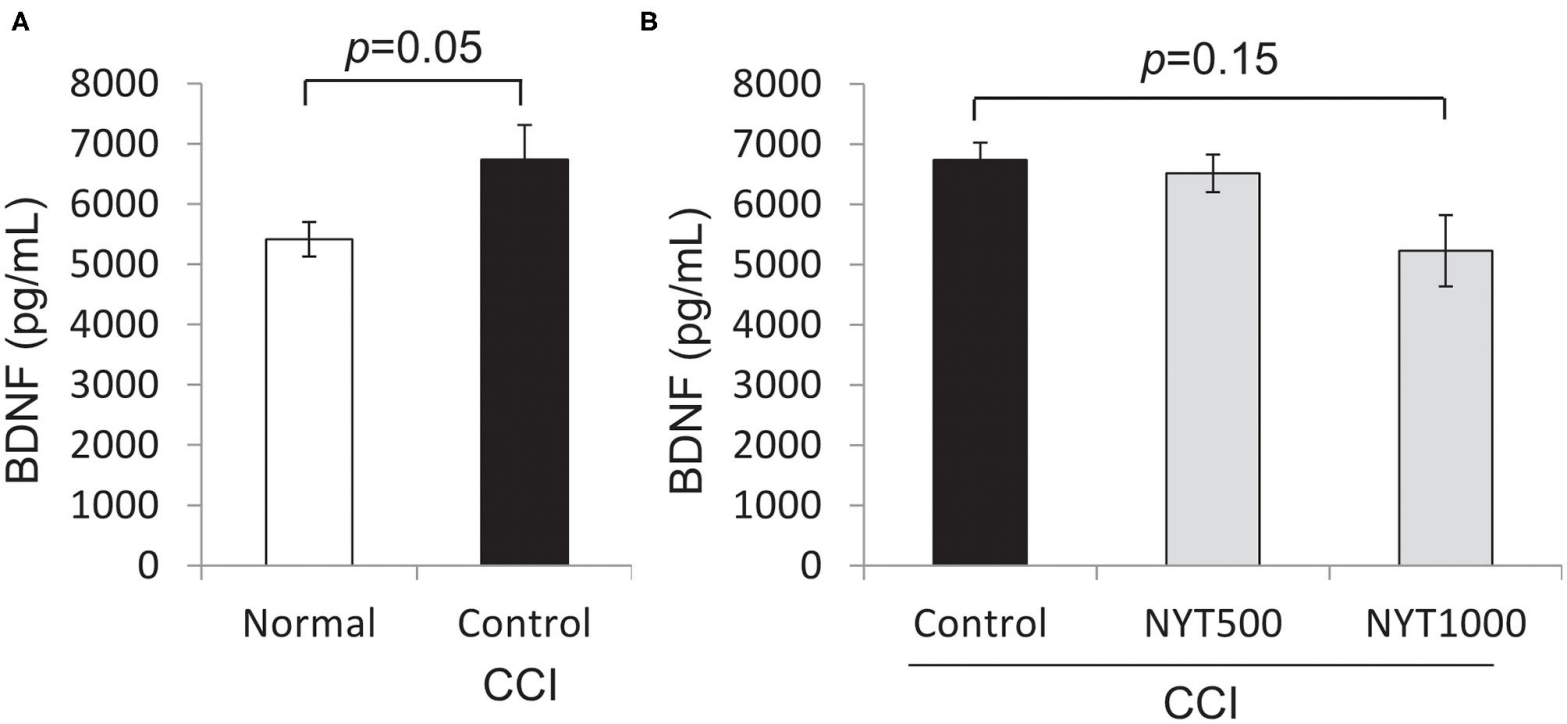
Effects of NYT on BDNF protein expression in serum. BDNF protein levels in serum. The black column group was administered water (Control); the gray column group was administered NYT (NYT500/NYT1000). The white column group comprised normal rats (Normal). **(A)** Normal vs CCI. **(B)** CCI vs NYT. Each column shows the mean ± S.E. of 7–8 mice. *p-*values evaluated using *t*-test and Dunnett's test.

BDNF is an important marker and modulator of neural activity and N-methyl-D-aspartate receptor-dependent neuronal plasticity in ascending and descending pain transmission pathways (14). In animals, BDNF and its tropomyosin receptor kinase B receptor were shown to be increased in models of bladder inflammation and nerve injury (15, 16). Furthermore, when BDNF was neutralized by the anti-BDNF antibody or tropomyosin receptor kinase B receptor, mechanical allodynia (17) and thermal hyperalgesia were alleviated (18). Based on these findings, it was suggested that BDNF contributes to pain sensitivity and intensity. In a clinical study, the serum BDNF level was significantly higher in patients with pain of endometriosis than in patients without pain (19). Using serum BDNF, we investigated whether BDNF is involved in the action mechanism of this study. Serum BDNF levels were higher in untreated CCI rats than in normal rats (*p* = 0.05, by Student's *t*-test), but decreased after the repeated administration of NYT (1,000 mg/kg, *p* = 0.15, by Dunnett's test) (Figure 3).

### Effects of NYT on Corticosterone Protein Expression in Serum

**Figure 4:**
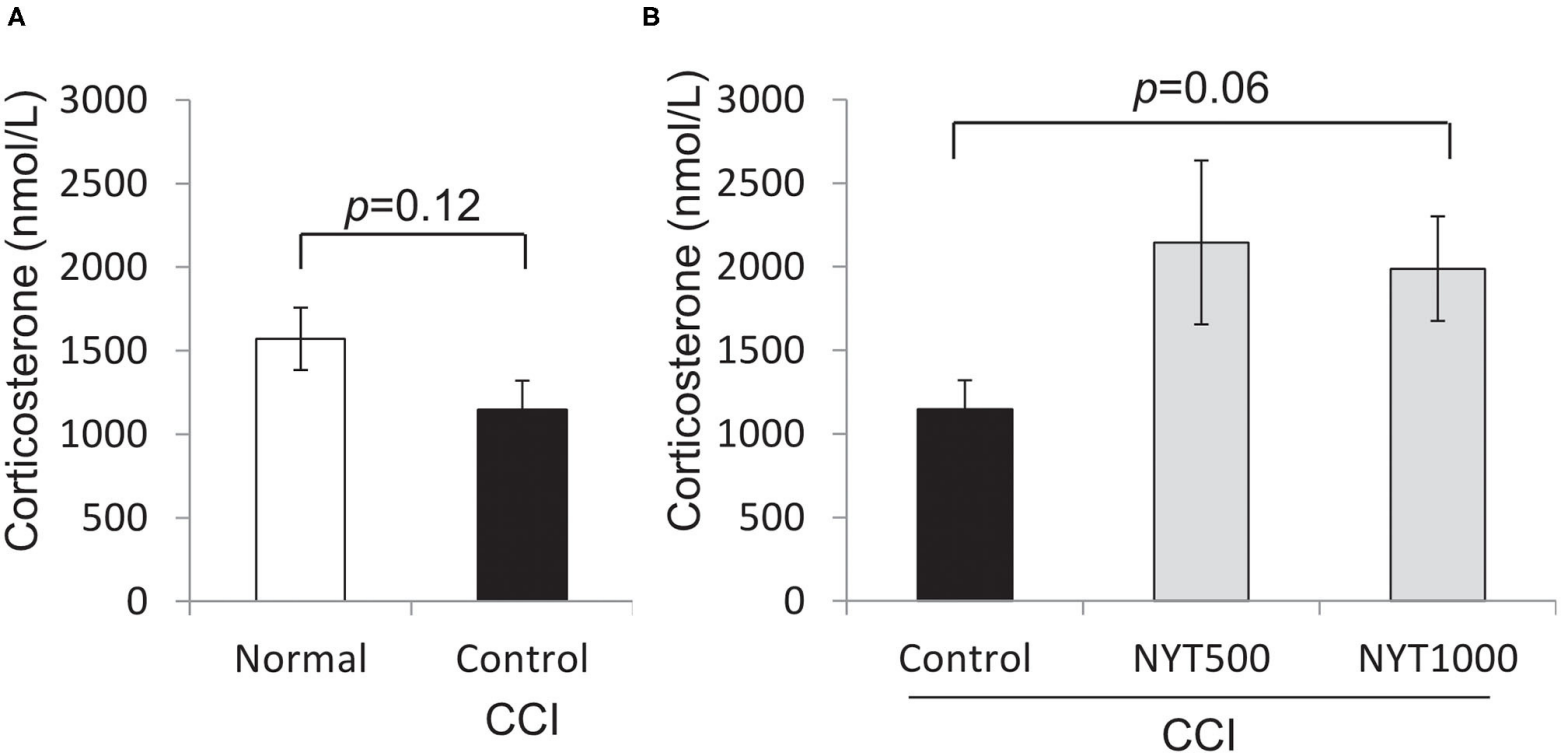
Effects of NYT on corticosterone protein expression in serum. Corticosterone protein levels in serum. The black column group was administered water (Control); the gray column group was administered NYT (NYT500/NYT1000). The white column group comprised normal rats (Normal). **(A)** Normal vs CCI. **(B)** CCI vs NYT. Each column shows the mean ± S.E. of 8 mice. *p*-values evaluated using *t*-test and Steel test.

Glucocorticoid hormones are regarded as “stress hormones” because they typically increase in response to environmental challenges (20–23). Corticosterone, the primary glucocorticoid in some vertebrates, is released following the activation of the hypothalamic-pituitary-adrenal (HPA) axis. Stress negatively affects hippocampal neurogenesis and plasticity through the activation of the HPA axis, resulting in the increased production of corticosterone and development of depressive-like symptoms (24). Since pain may activate the HPA axis (25, 26), we investigated whether our results were a consequence of the activation of the HPA axis in response to chronic pain as a stressful stimulus, and measured corticosterone serum levels in normal rats and CCI rats. In untreated CCI rats, serum corticosterone levels were lower (*p* = 0.12, by Student'*t*-test) than those in normal rats, and increased after the repeated administration of NYT (1,000 mg/kg, *p* = 0.07, by Steel test) (Figure 4).

## Discussion

We herein evaluated the efficacy of NYT to prevent neuropathic pain in CCI rats. Mechanical allodynia and spontaneous pain were significantly induced by CCI surgery and inhibited by NYT. These results are consistent with clinical findings. In addition, repeated administration of NYT decreased blood BDNF whereas blood corticosterone increased.

This study was focused on BDNF, which has been reported as a pain-related factor. A positive correlation between the blood and brain-tissue BDNF levels in rats has been reported (27), suggesting that the blood BDNF level reflects the brain-tissue BDNF level. In our study, the blood BDNF was measured expecting that the blood BDNF level reflects the brain-tissue BDNF level. In a preceding study, the BDNF level after CCI increased by 45% in the thalamus and by 27% in the midbrain (*p* > 0.05) (28). In addition, it has been reported that the BDNF level in CCI markedly increased in the periaqueductal gray matter (PAG), which is the important pain control center on the spinal cord (29), and the thalamic nucleus is decisively involved in down regulation of harmful mechanical and heat-inducing reactions (30). These reports concerning an increase in cerebral BDNF in CCI and the increase in serum BDNF in CCI in this study were consistent, suggesting that an NYT-induced decrease in the BDNF level is a part of the mechanism of the analgesic effect. On the other hand, the mechanism in the CCI model is analyzed mainly in the spinal cord and BDNF expression in the spinal cord is very important. However, the correlation between spinal cord BDNF and serum BDNF is unclear and investigation of BDNF expression in the spinal cord and upstream factors is necessary to more closely analyze the mechanism.

When the serum corticosterone level was measured in rats influenced by chronic pain, it was lower than that in normal rats. It has been reported that in an SD rat continuous stress model, a significant increase was observed on day 1, the level gradually decreased from day 2, and no significant difference from the level in the control was noted on day 5 (31), suggesting that the corticosterone level rises in response to acute stress, but responsiveness to stress worsens through subsequently becoming chronic. Our study demonstrated that NYT elevated the corticosterone level. In a study on the levels of the ingredients of NYT, intraperitoneal administration of Ginseng saponin significantly increased plasma corticosterone in normal rats, but Ginsenoside Rg1 decreased an increased serum corticosterone level in a depression model in another study (useful for behavior of depression). Based on the ingredient levels, NYT may influence the blood corticosterone level in the normal body, but considering the results of the depression model described above and our model, it is also likely to return an increased/decreased serum corticosterone to the normal state. In addition, NYT and a component crude drug, onji (polygala root), has an antidepressant effect (32, 33). Chenpi (Citrus Unshiu Peel) has been demonstrated to exhibit an effect on serotonin in the nervous system (34). Our study suggested that normalization of responsiveness to stress leads to the analgesic effect. Stress responsiveness and the mechanism of pain relief are interesting and remain as issues in the future.

Multiple mechanisms resulting from sciatic nerve injury have demonstrated that demyelination, ectopic discharge, and macrophage infiltration are closely associated with the development of neuropathic pain behaviors (35, 36). Myelinated A fibers at the distal CCI stumps of the sciatic nerve undergoing nerve demyelination increase ectopic discharge, which is regarded as an injury-induced electrophysiological characteristic (37, 38). On the other hand, a crude drug composing NYT, Citrus Unshiu Peel, has been reported to influence demyelination in the mouse brain (39), suggesting that NYT relives pain only through myelination. In the future, We want to investigate the mechanisms of these.

## Data Availability Statement

All datasets generated for this study are included in the article/supplementary material.

## Ethics Statement

The animal study was reviewed and approved by the Experimental Animal Care Committee of Kracie Pharma, Ltd. Written informed consent was obtained from the owners for the participation of their animals in this study.

## Author Contributions

RiT, SM, L-KH, NF, and RyT performed the experiments and analyzed the data. SM, NF, and RyT initiated and supervised the study. RiT, SM, NF, and L-KH designed experiments. RiT and L-KH wrote the manuscript. All authors contributed to the article and approved the submitted version.

## Conflict of Interest

All authors are employees of Kracie Pharma, Ltd.
